# Serum tenascin-C predicts resistance to steroid combination therapy in high-risk Kawasaki disease: a multicenter prospective cohort study

**DOI:** 10.1186/s12969-021-00562-w

**Published:** 2021-06-05

**Authors:** Yukako Yoshikane, Yoshiaki Okuma, Tatsuki Miyamoto, Junichi Hashimoto, Ryuji Fukazawa, Taichi Kato, Atsuhito Takeda, Kenji Suda, Takeji Matsushita, Michiaki Hiroe, Kyoko Imanaka-Yoshida

**Affiliations:** 1 Department of Pediatrics, Faculty of Medicine, Fukuoka University, 7-45-1 Nanakuma, Jonan, Fukuoka, 814-0133 Japan; 2 Department of Pediatrics, National Center for Global Health and Medicine, Tokyo, Japan; 3 Department of Pediatrics, Nippon Medical School, Tokyo, Japan; 4 Developmental Pediatrics, Nagoya University Graduate School of Medicine, Nagoya, Aichi Japan; 5 Department of Pediatrics, Hokkaido University Graduate School of Medicine, Sapporo, Hokkaido Japan; 6 Department of Pediatrics and Child Health, Kurume University School of Medicine, Fukuoka, Japan; 7 Department of Cardiology, National Center for Global Health and Medicine, Tokyo, Japan; 8 Department of Pathology and Matrix Biology, Mie University Graduate School of Medicine, Tsu, Mie Japan

**Keywords:** Kawasaki disease, Tenascin C, Biomarkers, Prospective study, Kobayashi score, High-risk, Resistant, IVIG, Steroids, Prednisolone

## Abstract

**Background:**

Tenascin-C (TN-C) is an extracellular matrix glycoprotein related to tissue inflammation. Our previous retrospective study conducted in 2016 revealed that the serum tenascin-C level was higher in patients with Kawasaki disease (KD) who were resistant to intravenous immunoglobulin (IVIG) and developed coronary artery lesions (CALs). The present study is a prospective cohort study to assess if the serum level of tenascin-C could be used as a novel biomarker to predict the risk of resistance to initial treatment for high-risk patients.

**Methods:**

A total of 380 KD patients were registered and provided serum samples for tenascin-C measurement before commencing their initial treatment. Patients who did not meet the inclusion criteria were excluded from analysis; of the 181 remaining subjects, there were 144 low-risk patients (Kobayashi score: ≤4 points) and 37 high-risk patients (Kobayashi score: ≥5 points). The initial treatments for low-risk patients and high-risk patients were conventional therapy (IVIG with aspirin) and prednisolone combination therapy, respectively. The patient clinical and laboratory data, including the serum tenascin-C level, were compared between initial treatment responders and non-responders.

**Results:**

In the low-risk patients, there was no significant difference in the median levels of serum tenascin-C between the initial therapy responders and non-responders. However, in the high-risk patients, the median serum tenascin-C level in initial therapy non-responders was significantly higher than that in initial therapy responders (175.8 ng/ml vs 117.6 ng/ml).

**Conclusions:**

Serum tenascin-C could be a biomarker for predicting the risk of high-risk patients being non-responsive to steroid combination therapy.

**Trial registration:**

This study was a prospective cohort study. It was approved by the ethics committee of each institute and performed in accordance with the Declaration of Helsinki.

## Background

Kawasaki disease (KD) is an acute systemic vasculitis of unknown etiology that occurs in early childhood [1]. Coronary artery lesions (CALs) are the most critical complication of KD; they can lead to myocardial ischemia, infarction, or even sudden death in adulthood. Treatment with a high dose of intravenous immunoglobulin (IVIG) is the most effective evidence-based therapy for the acute phase of KD, and it significantly reduces the rate of CALs [2, 3]. However, approximately 20% of patients with KD still have persistent or recrudescent fever after initial IVIG treatment [4], and IVIG resistance is a major risk factor for CAL development [5, 6]. Some scoring systems can predict initial IVIG resistance in patients at the time of KD diagnosis [7–9]. Specifically, the Kobayashi scoring system predicts patients at high risk of IVIG resistance with 76% sensitivity and 80% specificity among Japanese individuals, so it is widely used in Japan [8]. Japanese guidelines recommend the use of steroid combination therapy for these high-risk patients [10]. The RAISE study showed that additional prednisolone treatment reduced the non-response to IVIG and decreased the occurrence of CALs in high-risk KD patients [11, 12]. Intravenous methylprednisolone plus IVIG also had a positive effect [13, 14]. However, according to a Japanese national survey, the CAL rate has recently remained at approximately 2.5% [15]. Thus, the currently applied stratification of severe cases who do not respond to initial steroid combination therapy is still insufficient. Additionally, the accuracies of scoring systems used in other countries are not high [16, 17]. Therefore, efforts are ongoing to find a simple biomarker with which to stratify patients with KD [18, 19].

Tenascin-C (TN-C) is a large extracellular matrix glycoprotein [20] that is sparsely expressed in normal tissue but is upregulated in association with tissue injury and inflammation [21–23]. It has diverse functions in regulating cell behavior during inflammation and tissue repair in many pathological processes [24–26]. Because of its specific expression, serum TN-C is used as a biomarker for assessing disease activity and predicting prognosis in various cardiovascular diseases, such as dilated cardiomyopathy [27], acute myocardial infarction [28], aortic aneurysm/dissection [29, 30], and coronary atherosclerosis [31]. In 2015, we showed that TN-C was highly expressed in the vessel walls of *Candida*-induced KD vasculitis model mice [32]; we thus proposed that TN-C may be involved in the process of CAL formation.

Our previous retrospective study suggested that the serum TN-C level may be a promising biomarker for predicting the risk of CAL occurrence and IVIG resistance in patients during the acute phase of KD, with an accuracy comparable to that of the Kobayashi score [33]. In the present study, we examined whether the serum TN-C level could be used as a biomarker for predicting resistance to first line therapy, even in KD patients stratified as high risk.

## Methods

### Subjects

We conducted a multicenter prospective study and enrolled 380 KD patients who were hospitalized across 17 hospitals in Japan (Japan Community Health Care Organization Hokkaido Hospital; KKR Sapporo Medical Center; NTT East Sapporo Hospital; Tenshi Hospital; Teine Keijinkai Hospital; Japanese Red Cross Kitami Hospital; Nikko Memorial Hospital; Kushiro Red Cross Hospital; National Center for Global Health and Medicine; Japanese Red Cross Medical Center; Toho University Medical Center Oomori Hospital; Showa University Hospital; Tokai University Oiso Hospital; Gunma Children’s Medical Center; Japanese Red Cross Nagoya Daiichi Hospital; Fukuoka Children’s Hospital; and Fukuoka University Chikushi Hospital) between April 2011 and March 2015. We diagnosed KD in accordance with the Japanese diagnostic guidelines for KD [34]. The study design was approved by each institution’s ethics committee. Written informed consent was obtained from the participants or their parents/guardians.

### Protocols

The patients were categorized into two groups on the basis of their Kobayashi score: a group of low-risk patients who scored ≤4 points, and a group of high-risk patients who scored ≥5 points at the time of diagnosis. All KD patients were treated in accordance with the guidelines from the Japanese Pediatric Cardiology and Cardiac Surgery 2012 [35].

Low-risk patients received the conventional treatment consisting of 2 g/kg of IVIG with aspirin. High-risk patients, in accordance with the RAISE study protocol, received conventional treatment plus 2 mg/kg of prednisolone per day [11]. Prednisolone was administered by intravenous injection in three divided doses for over 5 days. If the patient’s fever resolved, the prednisolone administration route was changed from intravenous to oral. When the concentration of C-reactive protein normalized (≤5 mg/L), the prednisolone dose was tapered off over 15 days. During the prednisolone administration, famotidine, which is a histamine-2 receptor antagonist, was co-administered (1 mg/kg/day). Aspirin treatment began with a dose of 30 mg/kg per day. After patients became afebrile, the aspirin dose was reduced to 3–5 mg/kg per day; aspirin was administered for over 2–3 months after fever onset. For initial therapy non-responders (persistent fever for over 24 h after the initial treatment was terminated), an additional treatment was added in accordance with the strategy of each institute.

Two-dimensional echocardiograms were performed upon admission, on days 10–14, and on days 30–40 from the onset of KD, and the intraluminal diameters of coronary artery segments were measured at each institute. The presence of CALs was diagnosed on the basis of the z-scores of the left main trunk coronary artery (LMT), the proximal left anterior descending coronary artery (LAD), and the right coronary artery (RCA) [36]. CALs were defined on the basis of the z-scores as follows: z-score of < 2.0, no involvement; z-score of ≥2.0 to < 2.5, dilation; z-score of ≥2.5 to < 10, aneurysm; and z-score of ≥10, giant aneurysm.

### Measurement of serum TN-C levels

Serum samples were obtained to measure the serum TN-C levels before initial treatment and two or 3 days after initial treatment was begun. Blood samples were sent to the National Center for Global Health and Medicine, where serum TN-C levels were measured by performing an enzyme-linked immunosorbent assay using the Human TN-C Large (FN III-Japan). Medical, demographic, and laboratory data were collected from all cases upon admission.

### Statistical analysis

All analyses were performed using SPSS software, version 20 (SPSS Japan, Tokyo, Japan). Data are presented as the median and data range (minimum to maximum) for continuous variables or as the percentage of patients for each categorical variable. A series of group comparisons were conducted using *t*-tests for numerical data with a normal distribution or Mann-Whitney U tests for data lacking a normal distribution. The Kolmogorov-Smirnov algorithm was used to identify whether variables had a normal distribution. For all comparisons, differences with a *p*-value of < 0.05 were considered statistically significant.

## Results

### Patient characteristics

**Fig. 1:**
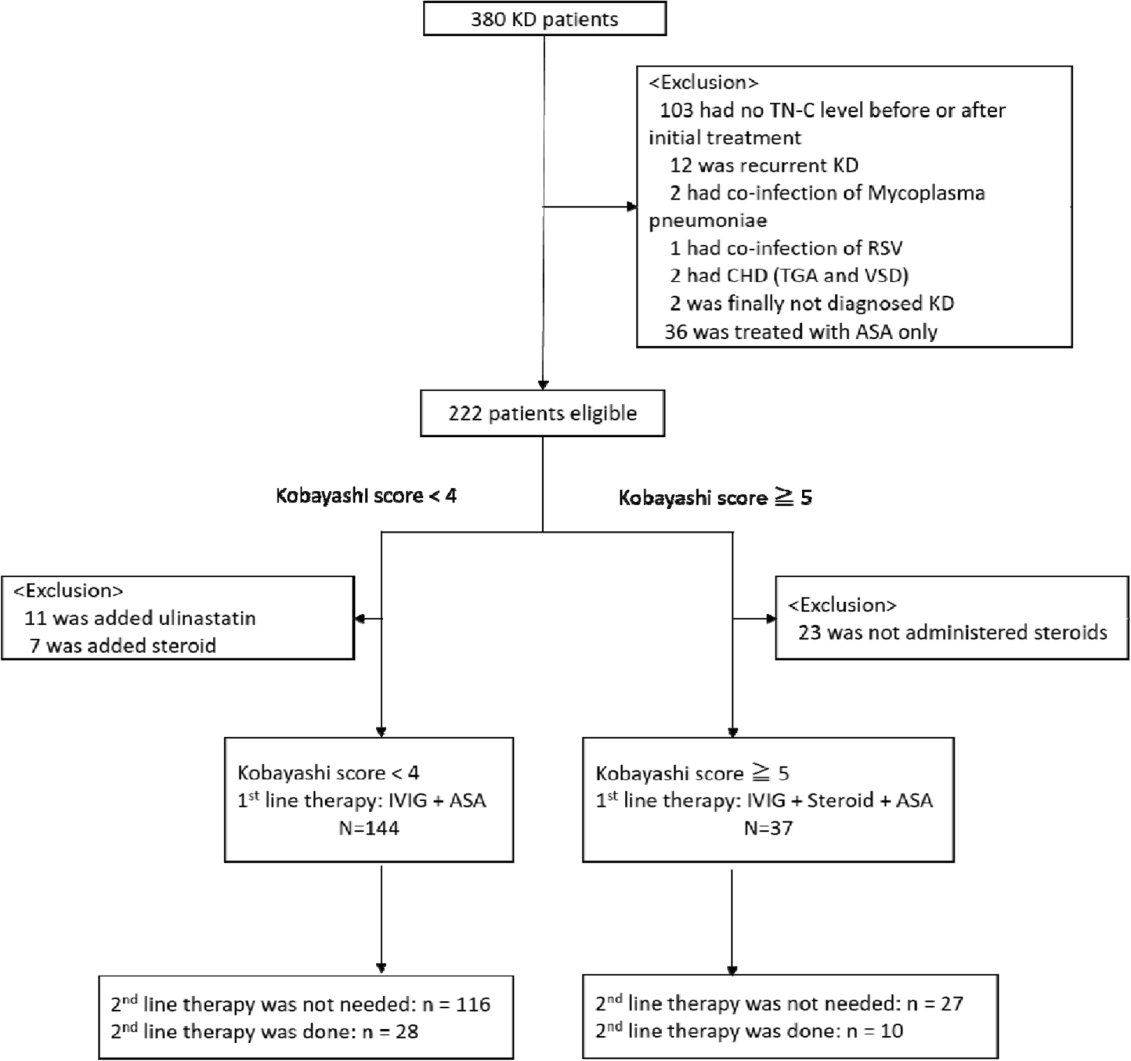
Study population. Eligible patients were classified into four groups: low-risk initial treatment-responsive group (*n* = 116), low-risk initial treatment-resistant group (*n* = 28), high-risk initial treatment-responsive group (*n* = 27), and high-risk initial treatment-resistant group (*n* = 10)

In total, 380 patients were diagnosed with KD upon admission to participating institutions during the study period. We first excluded the patients for whom there was no available TN-C data from either before or after their initial treatment (*n* = 103), patients who had a recurrent case (*n* = 12), those with a concurrent infection (*n* = 3), those with underlying congenital heart disease (*n* = 2), those who were not finally diagnosed with KD (n = 2), and those who did not receive IVIG treatment (*n* = 36) (Fig. 1). The remaining 222 patients were categorized into two groups on the basis of whether their Kobayashi score was ≤4 points (low-risk patients, *n* = 162) or ≥ 5 points (high-risk patients, *n* = 60) at the time of diagnosis (Table 1).

**Table 1:** Scoring systems that predict initially IVIG-resistant patients

Kobayashi Score (Cut off: ≥5 points; Sensitivity 76%、Specificity 80%)		
Risk Factor		Points
Illness days at diagnosis	< 4 days	2
Serum sodium level	< 133 mmol/l	2
AST	≥ 100 IU/l	2
Neutrophil rate	≥ 80%	2
CRP	≥ 10 mg/dl	1
Platelet count	≤30.0 × 104/mm3	1
Age at diagnosis	≤12 month	1

Regarding the low-risk patients, we further excluded those whose treatment included added ulinastatin (*n* = 11) or steroids (*n* = 7). This left 144 enrolled patients who were administered IVIG as a first-line therapy. Among them, 116 (80.6%) patients responded to the IVIG-treatment and did not require a second-line therapy (low-risk initial treatment-responsive group), while 28 patients were resistant to the IVIG and did require a second-line therapy (low-risk initial treatment-resistant group). Regarding the high-risk patients, we excluded those who were not administered steroids (*n* = 23). This left 37 patients who were administered IVIG+steroid as a first-line therapy. Among them, 27 (73.0%) patients responded to the IVIG+steroid and did not require a second-line therapy (high-risk initial treatment-responsive group), while 10 patients did not respond to the IVIG+steroid and did require a second-line therapy (high-risk initial treatment-resistant group).

The baseline characteristics of the high-risk patients and low-risk patients are shown in Tables 2 and 3, respectively. In both the low- and high-risk groups, there were no significant differences in terms of age, sex, and laboratory data between the initial treatment-responsive group and the initial treatment-resistant group.

**Table 2:** Characteristics and data of high-risk patients in the initial treatment (IVIG+steroid)-responsive and initial treatment-resistant groups

	IVIG +steroid responder group	IVIG +steroid resistant group	*p*-value
Number	27	10	
Age (months)	42 [11–80]	44 [9–86]	0.880
Male gender, n (%)	17 (63.0)	8 (80.0)	0.285
Illness day at diagnosis	4 [2–8]	3 [2–7]	0.220
Kobayashi score	6 [5–10]	6 [5–10]	0.216
< Laboratory data before 1st line therapy >			
TN-C, ng/mL	117.6 [35.0–324.8]	175.8 [80.4–380.9]	0.037
WBC, × 10^3^/μL	14.8 [6.6–33.2]	18.6 [6.9–36.8]	0.242
Neutrophils, %	83 [60–95]	88 [68–94]	0.191
Platelets, × 10^4^/mL	26.2 [13.1–59.4]	23.9 [13.5–36.6]	0.555
CRP, mg/dL	10.0 [2.5–24.0]	10.1 [5.2–21.7]	0.853
Albumin, g/dL	3.6 [2.7–4.1]	3.6 [2.8–4.4]	0.801
T-bilirubin, mg/dL	0.7 [0.3–5.5]	1.4 [0.5–4.6]	0.391
AST, IU/L	57 [20–787]	551 [25–2725]	0.013
ALT, IU/L	83 [8–937]	518 [9–1435]	0.067
Sodium, mEq/L	133 [127–137]	132 [128–135]	0.578

*TN-C* tenascin-C, *WBC* white blood cell, *CRP* C-reactive protein, *AST* aspartate aminotransferase, *ALT* alanine aminotransferase

* The Kobayashi score [8] was ≥5 points in all cases

* In all cases, the first-line therapy was IVIG, prednisolone, and aspirin

**Table 3:** Characteristics and data of low-risk patients in the initial treatment (IVIG)-responsive and initial treatment-resistant groups

	IVIG-responder group	IVIG-resistant group	*p* value
number	116	28	
Age in month	29.5 [4–130]	22 [4–107]	0.352
Male gender, n (%)	56 (48.3)	13 (46.4)	0.861
Illness day at diagnosis	5 [2–10]	5 [3–8]	0.436
Kobayashi score	2 [0–4]	3 [0–4]	0.030
< Laboratory data before IVIG >			
TN-C, ng/mL	106.6 [29.1–449.6]	113.5 [46.6–483.4]	0.432
WBC, × 10^3^/μL	13.0 [6.1–32.3]	12.7 [6.3–22.0]	0.435
Neutrophil, %	66 [24–91]	67 [26–88]	0.418
Platelet, ×10^4^/mL	33.3 [16.6–53.3]	32.9 [19.4–62.5]	0.612
CRP, mg/dL	6.7 [1.3–31.4]	8.8 [1.5–20.0]	0.190
Albumin, g/dL	3.6 [2.6–4.8] *n* = 115	3.6 [2.6–4.3]	0.998
T-bilirubin, mg/dL	0.5 [0.1–3.1] *n* = 114	0.6 [0.2–3.4]	0.064
AST, IU/L	34 [15–298]	33 [18–236]	0.677
ALT, IU/L	19 [5–442]	24 [8–237]	0.608
Sodium, mEq/L	136 [127–143]	135 [127–138]	0.093

*TN-C* tenascin-C, *WBC* white blood cell, *CRP* C-reactive protein, *AST* aspartate aminotransferase, *ALT* alanine aminotransferase

* The Kobayashi score [8] was < 5 points in all cases

* In all cases, the first-line therapy was IVIG and aspirin

### Serum TN-C levels

**Fig. 2:**
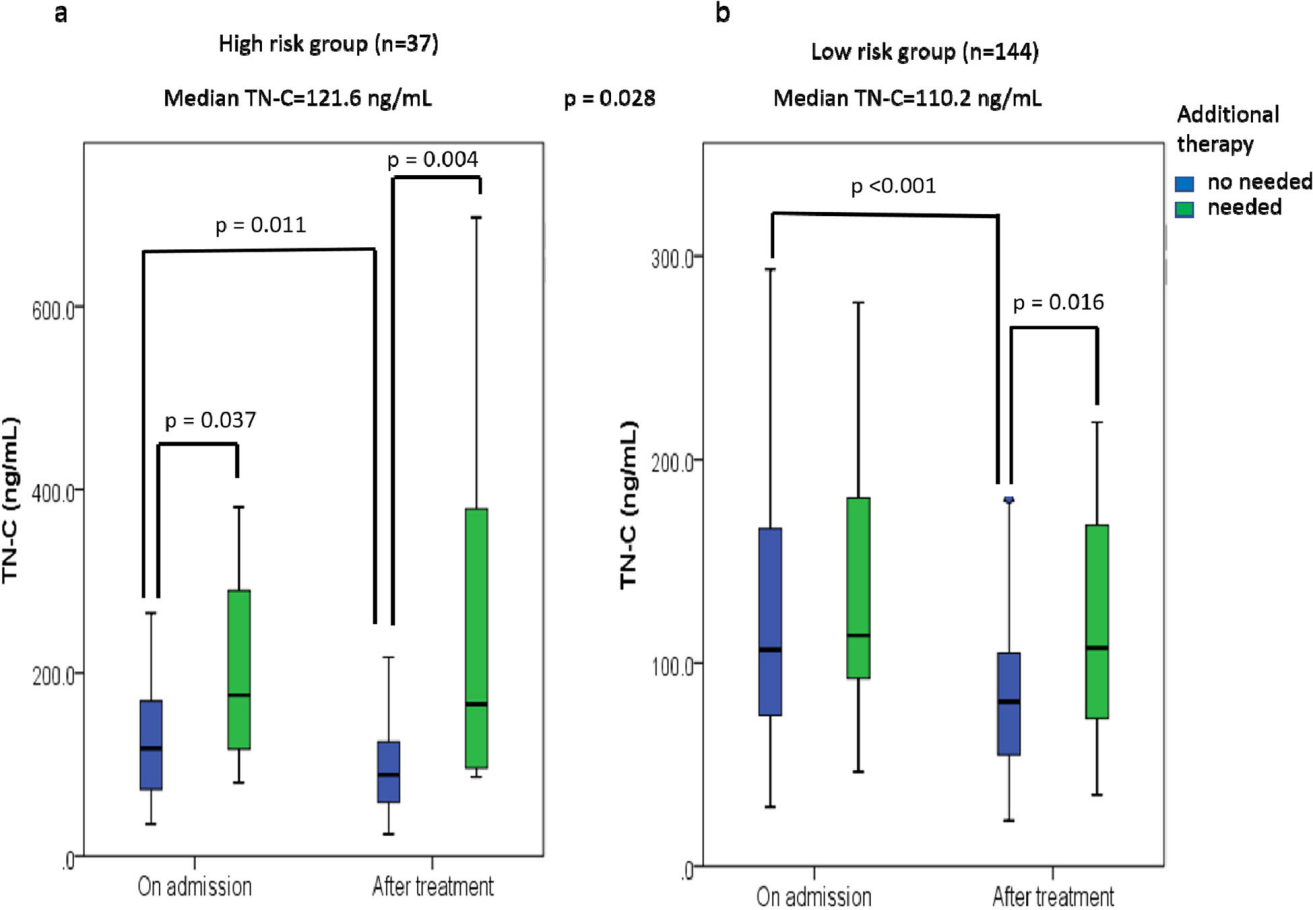
TN-C levels in high- and low-risk patients. (**a–b**) Median levels of TN-C in high-risk (**a**) and low-risk (**b**) patients. “On admission” means “before initial treatment”, and “after treatment” means “after initial treatment”

First, we compared the serum TN-C levels on admission between the high-risk patients and the low-risk patients. The median level of TN-C for the high-risk patients was significantly higher than that of the low-risk patients (median: 121.6 [35.0–380.9] ng/ml vs. 110.2 [29.1–293.6] ng/ml, *p* = 0.028) (Fig. 2).

Among the high-risk patients, the median TN-C level on admission for the first-line treatment-resistant group (IVIG+ASA + steroid) was significantly higher than that of the first-line treatment-responsive group (median: 175.8 [80.4–380.9] ng/ml vs. 117.6 [35.0–324.8] ng/ml, *p* = 0.037) (Fig. 2a). After the first line-treatment was initiated, the level of TN-C was significantly reduced in the initial treatment-responsive group (median: 117.6 [35.0–324.8] ng/ml to 88.7 [23.8–263.3] ng/ml, *p* = 0.011), whereas no significant change in TN-C level was found for the initial treatment-resistant group (median: 175.8 [80.4–380.9] ng/ml to 166.1 [86.2–696.2] ng/ml, *p* = 0.878). Hence, the post-first treatment median TN-C level of the patients who required a second-line treatment was significantly higher than that of the patients who did not need additional treatment (median: 166.1 [86.2–696.2] ng/ml vs. 88.7 [23.8–263.3] ng/ml, *p* = 0.004).

Among the low-risk patients, no significant difference in the level of TN-C upon admission was found between the initial treatment-responsive group and the initial treatment (IVIG+ASA)-resistant group (median: 106.6 ng/ml [29.1–293.6] vs. 113.5 [46.6–277.4] ng/ml, *p* = 0.432) (Fig. 2b). As in the high-risk patients, the first-line treatment significantly reduced the TN-C level in the initial treatment-responsive group of low-risk patients (median: 106.6 [29.1–293.6] ng/ml to 81.1 [22.4–181.4] ng/ml, *p* < 0.001), whereas no significant change was found in the initial treatment-resistant group of low-risk patients (median: 113.5 [46.6–277.4] ng/ml to 107.3 [35.1–218.5] ng/ml, *p* = 0.212). Again, the post-first-line treatment TN-C levels of the initial treatment-resistant patients were significantly higher than those of the group who did not require additional treatment (median: 107.3 [35.1–218.5] ng/ml vs. 81.1 [22.4–181.4] ng/ml, *p* = 0.016).

### Coronary artery lesions

Among the 37 high-risk patients, three (8.1%) had coronary aneurysms (z-score: ≥2.5). The serum TN-C level upon admission was not significantly different between the CAL-positive and CAL-negative groups of high-risk patients (119.5 ± 33.0 ng/ml vs. 120.4 ± 75.3 ng/ml, *p* = 0.291). Among the 144 low-risk patients, there were seven (4.9%) who had coronary aneurysms (z-score: ≥2.5). Similarly, the serum TN-C level upon admission in the low-risk patients was not significantly different between the CAL-positive and CAL-negative groups (150.4 ± 67.5 ng/ml vs. 110.2 ± 43.6 ng/ml, *p* = 0.835).

## Discussion

The present multicenter prospective study revealed that the serum TN-C level can be a useful biomarker for treatment selection in the acute phase of KD. The serum level of TN-C upon admission of the patients categorized as high-risk on the basis of their Kobayashi score was significantly higher than that of the low-risk patients. This finding is consistent with the results of our previous retrospective study; together, these results suggest that the serum TN-C level alone could be a biomarker for identifying high-risk patients, comparable with the Kobayashi score [8].

Histologically, coronary arteritis begins 6–8 days after KD onset and is characterized by inflammation consisting of a marked accumulation of monocytes/macrophages [37]. TN-C is expressed in the areas where inflammatory lesions form on coronary arteries during the acute stage of KD, and the intensity of its expression correlates with the degree of inflammation [38]. Therefore, a high serum level of TN-C may reflect severe inflammation.

Japanese guidelines allow the stratification of KD patients by scoring systems of predicted IVIG resistance, treating patients classified as low-risk with IVIG and those identified as high-risk with a steroid/conventional IVIG combination as the first-line therapy. Recently, however, unresponsiveness to this treatment protocol has become a major problem. In our present study, 28 out of the 114 low-risk patients were not responsive to their initial therapy of IVIG, and 10 out of the 37 high-risk patients were not responsive to their initial treatment with steroid combination therapy, such that they all required additional therapies. Importantly, in the high-risk patients, the serum TN-C levels upon admission of the initial treatment-resistant patients were significantly higher than those of the initial treatment-responsive patients. This finding suggests that the stratification of KD severity by Kobayashi score has limitations and that the TN-C level could be used as a biomarker for identifying those at greater risk of resistance to first-line treatment among patients classified as high-risk on the basis of their Kobayashi score.

Jone et al. demonstrated that the use of IVIG plus infliximab as initial therapy reduces the need for additional therapy in KD patients presenting with CALs [39]. Hamada et al. demonstrated that treatment with IVIG plus cyclosporine was safe and effective for favorable coronary artery outcomes in high-risk KD patients [40]. Notably, KD patients who are predicted to be at high risk require the use of a more potent treatment in addition to or instead of treatment with steroids/IVIG.

In the low-risk patients, the TN-C level upon admission was not significantly different between the initial treatment-responsive and initial treatment-resistant patients, suggesting that the TN-C level may not be a valid predictor of IVIG-resistance in this group. Thus, the TN-C level may be useful as a predictive biomarker only in the high-risk cases.

In both high-risk and low-risk patients, the first-line therapy significantly reduced the TN-C levels in the initial treatment-responsive patients but did not do so in the initial treatment-resistant patients. The patients with higher TN-C levels after the initial treatment needed a second-line therapy. These findings suggest that the TN-C level reflects the effectiveness of the treatment and could be applied as a rationale for commencing additional therapy. Because steroids often mask the symptoms of inflammation, their use can make it difficult for physicians to accurately judge whether the inflammation has decreased or if further intervention is needed. On the basis of the findings of the present study, however, the TN-C level did not decrease in high-risk patients after the initial treatment. Thus, the TN-C level might not be masked by steroids and could be useful as an indicator of whether additional treatment is needed.

In the present study, there was no difference in the TN-C levels of patients with or without CALs. This could be because the number of CALs in our enrolled patients was too small for accurate evaluation. However, it is well known that treatment resistance is closely related to CAL onset [6–8], which suggests that there would likely have been a significant difference if our study sample size was larger. If the use of biomarkers, such as the TN-C level, could predict a severe course of disease, the ensuing application of more aggressive initial and/or additional treatment might be expected to reduce the incidence of CALs.

There are some limitations of this study. Our sample size might be insufficiently large to contain enough severe cases who need additional treatment. Among the enrolled subjects, there were 37 cases with high-risk patients, of which only 10 cases (5% of all subjects) did not respond to initial treatment with steroid combination therapy. Because only 5% of our subjects had such refractory cases, future studies on this topic should aim to recruit more subjects than were included here. In addition, a total of 199 patients (52%) were excluded because they failed to meet the inclusion criteria. However, there were no significant differences in age, Kobayashi score, or laboratory data between the group containing all 380 patients and the subset of 181 subjects who were included in our analysis. Thus, we think there was no bias introduced by applying our inclusion criteria.

## Conclusions

The serum level of TN-C could be used as a biomarker for predicting KD severity. Among high-risk patients, the early identification of severe cases who are resistant to steroid combination therapy could help to prevent CAL occurrence through the application of aggressive treatment strategies. It is expected that severity diagnosis using biomarkers such as the TN-C level will be added into the treatment guidelines for acute KD worldwide.

## Data Availability

Not applicable.

## Abbreviations

TN-C: Tenascin-C
KD: Kawasaki disease
IVIG: Intravenous immunoglobulin
CALs: Coronary artery lesions
